# Bayesian Classifier with Simplified Learning Phase for Detecting Microcalcifications in Digital Mammograms

**DOI:** 10.1155/2009/767805

**Published:** 2010-01-04

**Authors:** Imad Zyout, Ikhlas Abdel-Qader, Christina Jacobs

**Affiliations:** ^1^Department of Electrical and Computer Engineering, Western Michigan University, MI 49008, USA; ^2^Radiology Department, Bronson Methodist Hospital, Kalamazoo, MI 49007, USA

## Abstract

Detection of clustered microcalcifications (MCs) in mammograms represents a significant step towards successful detection of breast cancer since their existence is one of the early signs of cancer. In this paper, a new framework that integrates Bayesian classifier and a pattern synthesizing scheme for detecting microcalcification clusters is proposed. This proposed work extracts textural, spectral, and statistical features of each input mammogram and generates models of real MCs to be used as training samples through a simplified learning phase of the Bayesian classifier. Followed by an estimation of the classifier's decision function parameters, a mammogram is segmented into the identified targets (MCs) against background (healthy tissue). The proposed algorithm has been tested using 23 mammograms from the mini-MIAS database. Experimental results achieved MCs detection with average true positive (sensitivity) and false positive (specificity) of 91.3% and 98.6%, respectively. Results also indicate that the modeling of the real MCs plays a significant role in the performance of the classifier and thus should be given further investigation.

## 1. Introduction

Breast cancer is the most common cancer among US women and remains the number two cause of cancer death for US women. The recent figures and facts published in 2008 by the American Cancer Association [1] indicate that 1 in 8 US women are at risk of developing breast cancer in a life time and that 1 in 35 US women are expected to die from this disease [1]. One of the early stages of this disease is the manifestation of clustered microcalcifications and hence detection of such features can be a life saving mechanism in the war against breast cancer. MCs detection is a challenging task for both radiologists and computer-aided diagnostic (CAD) systems due to minute and ubiquitous nature of microcalcifications. Microcalcifications appear in a mammogram as bright regions of different shapes and sizes, and attain gray-level intensities and contrast that depend on their surrounding tissue. Generally, microcalcifications can be characterized as follows: (1) malignant microcalcifications tend to exist in the form of clusters of irregular shapes and sizes while benign microcalcifications tend to appear as isolated spots mostly with regular, almost round, shapes [2], (2) the sizes of microcalcifications are very small, varying from 0.1 mm to 1 mm with an average size of about 0.3 mm [2]. Moreover, in some cases of isolated or benign MCs, they can appear with sizes of less than 0.1 mm. Also, MCs present in a dense breast tissue mostly have low local contrast adding to the challenge of identifying them within the neighboring healthy tissue [2]. 

 Computer-aided-diagnosis technology for breast cancer is mainly intended to be a second opinion that provides the radiologists with a preliminary diagnosis results, which has been demonstrated to increase the detection sensitivity (number of detected breast cancer) by an average of 10% [3]. In a study by Freer and Ulissey [4] by which they used CAD technology to interpret 12,860 mammograms collected over a 12-month period, they reported that employing CAD techniques increased the sensitivity of breast cancer detection by 19.5%, improved early detection of malignant cancer by 5%, and no significant increase of the recall rate was noticed. Several commercial breast cancer CAD systems have been FDA approved and currently used by radiologists, examples are Image Checker (R2 technologies, USA) and second look (iCAD systems, Canada). Although most CAD systems achieved high sensitivity for detecting MCs and masses [5], they still provide low sensitivity for amorphous microcalcifications and other mammogram abnormalities such as architectural distortions [6]. Also, current CAD systems cannot be used as second reader because of the low specificity of the detection estimated at 1000 benign alarms for one cancer [6].

Boyer et al. [6] presented a questions/answers review on the use of CAD technology for breast cancer. Using results from several relevant studies they addressed two important questions: (1) can a CAD system replace the first or double reading? and (2) what is the role of CAD technology within the diagnosis process? Boyer reported that a CAD system neither can be used as a first reading nor as a double reading. Rather, CAD systems can be used as an additional step to a double reading scheme, thus increasing the sensitivity of breast cancer diagnosis.

In general, a CAD system for MCs detection can be modeled as a two stages system. The first stage is a preprocessing phase to enhance the visual appearance of MCs using variety of enhancement methods and background suppression techniques. Also, it is in this stage a detection of suspected regions can be accomplished. In literature, researchers have applied several image processing techniques for enhancing contrast in the region of microcalcifications such as global enhancement techniques (contrast stretching [7], histogram equalizing [8]), fixed and adaptive local enhancement [9], and region based enhancement aiming to adjust the contrast of ROIs in relation to their surround-ing areas [10]. In fact, standard image enhancement methods failed to achieve a satisfactory enhancement of MCs which motivated the researcher to develop feature based enhancement methods such as locating features by extracting local statistical attributes of ROIs [3], fuzzy techniques [11], and multiscale analysis methods [12, 13]. Other enhancement methods focused on background subtraction to increase the appearance of MCs such as highpass filtering using wavelet reconstruction [14], fractal modeling [15], and morphological operators [16]. 

Aiming to detect the presence of MCs, the second stage is generally composed of several image segmentation, feature extraction and classification techniques. 

Previous studies used standard image segmentation methods such as edge detection based methods [11] and region growing based approaches [3]. The fuzzy nature of clustered microcalcifications made its detection a challenging task forcing the researchers to integrate several tools of feature extraction and classifications to overcome this challenge. Most detection schemes make use of statistical based feature extraction methods such as high-order statistics (kurtosis and skewness) parameters as in [17, 18] and image modeling based methods [19, 20], spectral features based methods [4, 18, 21], and textural based methods [19, 22]. Supervised learning machine based methods are also commonly used for feature classification and MCs detection [19, 20, 22–26]. A typical learning machine based method consists of supervised training and classification stages. Examples of MCs detection using combined feature extraction and supervised learning methods are artificial neural network [19, 23] based classifiers, stochastic discriminators such as Bayesian classifier [19, 22, 24, 25]), support vector learning machines methods [20, 26], fuzzy based approaches [27], and meta-heuristic methods such as genetic algorithm (GA) [28]. A more comprehensive review of automated microcalcification detection can be found in [3, 11].

Stochastic Bayesian classifier has been successfully employed for solving pattern recognition problems in many applications [19, 22, 24]; a popular example is its use in multi-spectral remote sensing imagery [24]. In this work, we propose a novel framework that uses linear Bayesian classifier with simplified training stage for the segmenta-tion and detection of microcalcification clusters in digital mammograms. Our proposed framework can be considered a single stage detection scheme consisting of two phases, namely, feature extraction and feature classification. In the feature extraction stage, four feature-images of a mammogram are constructed. These feature-images are stacked to form a multi-dimensional feature vector for each pixel of the original mammogram. In the learning stage, the parameters of the classifier's decision function are estimated using synthetic model of MCs while in the classification stage, the processes of learning and testing are implemented. It is in this stage the breast tissue is classified into positive (MCs) or negative (healthy) via maximizing a posteriori probability method. We believe that this framework is novel for two reasons: (1) it uses the Bayesian classifier (BC) as first stage to segment MCs as opposed to previous work which applied BC as second stage to classify suspicious regions into MCs or background and (2) it presents an unsupervised training by which it synthesizes the training set of MCs class and accomplishes the learning phase in an efficient and simplified method that does not require large training data set which is usually created by a tedious manually pre-labeling process [19, 22]. Instead, we propose to create models of the real MCs and produce synthetic learning samples to estimate the classifier's decision function parameters. 

The remaining sections of this paper are organized as follows: related work and background on stochastic Bayesian classifiers and wavelet transform are briefly discussed in Section 2. Section 3 presents the details of the proposed detection method while the Experimental Results and Conclusions are presented in Sections 4 and 5, respectively.

## 2. Background

### 2.1. Bayesian Classifier

Bayesian classifier (BC) is a statistical method used for classification by maximizing a class a posteriori probability.

The application of Bayesian classifiers for pattern recognition assumes a prior knowledge of an analytical expression of the probability density functions of various classes. Using a sufficient statistic sample patterns of each class, one can properly estimate the necessary parameters of its density function. Mathematically, Bayes classifier [24] has a decision function of the form

(1) $$d_{j}\left(x\right)=p\left(x\;|\;\omega_{j}\right)p\left(\omega_{j}\right),\;j=1,2,\ldots ,M,$$

where *p*(*x* | *ω*_*j*_) is the conditional probability density function of *n*-dimensional feature vector *x* belonging to a class *ω*_*j*_, *P*(*ω*_*j*_) is the priori probability of class *ω*_*j*_, and *M* is the number of classes. Assuming the probability density functions (PDF) of the measured features are Gaussian [19, 22] then, the *n*-dimensional Gaussian density function can be expressed as

(2) $$p\left(x|\omega_{j}\right)=\frac{1}{{\left(2\pi \right)}^{n/2}{\left|C_{j}\right|}^{1/2}}\;e^{-(1/2){\left(x-m_{j}\right)}^{T}\;C_{j}^{-1}(x-m_{j})},$$

where **C**_*j*_ and **m**_*j*_ are the covariance matrix and the mean vector of class *ω*_*j*_, respectively. Also |**C**_*j*_| is the determinant of the matrix **C**_*j*_.

 Since the decision function given in (1) is monotonically increasing and because of the exponential nature of the Gaussian density function of the conditional probability *p*(*x* | *ω*_*j*_) [24], the decision function can be rewritten as

(3) $$\begin{array}{cc} d_{j}\left(x\right) & =\ln\;\;p\left(\omega_{j}\right)-\frac{n}{2}\ln\;\;\left(2\pi \right)-\;\frac{1}{2}\ln\;\;\left|C_{j}\right|\\ & \;-\frac{1}{2}\;\left[{\left(x-m_{j}\right)}^{T}C_{j}^{-1}\left(x-m_{j}\right)\right]. \end{array}$$

Since the term (*n*/2)ln (2*π*) is common for all classes and assuming that all classes are equally likely, the decision function *d*_*j*_(*x*) reduces to

(4) $$d_{j}\left(x\right)=-\;\frac{1}{2}\ln\;\;\left|C_{j}\right|-\frac{1}{2}\left[{\left(x-m_{j}\right)}^{T}C_{j}^{-1}\left(x-m_{j}\right)\right].$$

A feature vector **x** is assigned to a class with a minimum distance *d*_*j*_(*x*), [24].

### 2.2. Wavelet Transform

Over the last two decades, wavelet theory and its multiresolution analysis (MRA) ability [29] has been recognized as the most powerful tools in signal processing. Unlike Fourier analysis, multiresolution representa-tion of wavelet transform provides spatial-frequency localization which enables the analysis of both local and global features of the processed signal. Wavelet transforms are a set of basis functions derived by translation and dilation of a single function, the mother wavelet, *ψ* which has the general form of

(5) $$\psi_{a,b}\left(x\right)=\frac{1}{\sqrt{a}}\psi \left(\frac{x-b}{a}\right).$$

Equation (5) shows that the frequency and spatial resolution of the wavelet function *ψ*_*a*, *b*_ are functions of the translation and dilation parameters *b* and *a*, respectively. 

A special case of (5) is obtained when translation and dilation parameters are integers with a scaling parameter **a** as an integer of base 2, resulting in the dyadic wavelet transforms and leading to the construction of orthonormal wavelet basis *ψ*_*j*, *k*_:

(6) $$\psi_{j,k}\left(x\right)=2^{-j/2}\psi \left(2^{-j}x-k\right).$$

Moreover, MRA using wavelet transform is based on the existence of two unique functions called wavelet and scaling functions. The scaling function is defined as

(7) $$\varphi_{j,k}\left(x\right)=2^{-j/2}\varphi \left(2^{-j}x-k\right),$$

where the wavelet function is defined as given in (6).

**Figure 1:**
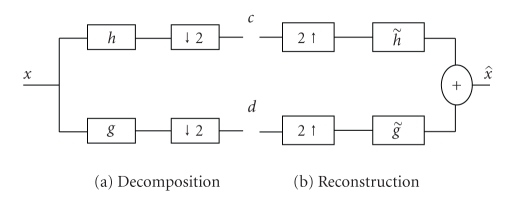
1D wavelet decomposition and reconstruction.

An efficient algorithm for computing discrete wavelet transform of a given discrete signal is introduced in [29] by which, each stage of wavelet decomposition process involves extracting an approximate (lowpass) version and a detail (highpass) version of the signal. This can be easily implemented using a set of finite impulse response (FIR) filter banks followed by sub sampling as shown Figure 1(a). 

The wavelet synthesis process as shown in Figure 1(b) is accomplished by first filtering the up-sampled *c* and *d* using the synthesis lowpass $\tilde{h}$ and highpass $\tilde{g}$ filters, respective-ly. Then, given that the set of analysis and synthesis filters satisfying perfect reconstruction conditions, an original signal $\hat{x}$ is obtained by adding the output of each filter, $\tilde{h}$ and $\tilde{g}$, [29]. Wavelet transforms are one dimensional in nature but easily extended to analyze 2-D discrete signals. Separable 2-D wavelet transform of an image is constructed by applying 1-D wavelet transform along the image rows and columns. The 2-D wavelet and scaling functions derived from 1D wavelet *ψ*(*x*) and scaling *φ*(*x*) functions, are expressed as

(8) $$\begin{array}{c} \varphi \left(x,y\right)=\varphi \left(x\right)\varphi \left(y\right),\\ \psi^{H}\left(x,y\right)=\varphi \left(x\right)\psi \left(y\right),\\ \psi^{V}\left(x,y\right)=\psi \left(x\right)\varphi \left(y\right),\\ \psi^{D}\left(x,y\right)=\psi \left(x\right)\psi \left(y\right), \end{array}$$

where *φ*(*x*, *y*) represents a 2-D separable lowpass filter applied along the horizontal *x* vertical *y* directions. *ψ* ^*H*^(*x*, *y*), *ψ* ^*V*^(*x*, *y*), *ψ* ^*D*^(*x*, *y*) are 2-D separable highpass filters extracting the signal details along the horizontal, vertical, and diagonal directions, respectively.

Variety of wavelet transforms have been proposed and used in the literature [24, 29, 30] in many applications. These transforms have different features such as regularity, number of vanishing moments, orthogonality, symmetry, and compact support. However, the selection of a wavelet transform with certain features is an application dependant.

### 2.3. Related Work

The Gaussian nature of the MCs gray-level distribution and their wavelet representation [21, 31] was used along with other textural features and with a Bayesian classifier to identify the true MCs [19, 22]. This Gaussian nature justifies the use of Bayesian classifier in microcalcification detection. Yu et al. [19] proposed a two stage scheme for detecting MCs by which they used wavelet based filtering and global thresholding to identify suspicious MCs regions. Then, they employed Bayes and back propagation neural network (BPNN) classifiers to identify MCs, that is, reduce the number of false signals resulted from wavelet filtering [14]. Also, they used statistical Markov random field (MRF) modeling along with other image processing techniques to extract primary and secondary features of suspicious MCs and to serve as inputs of the classifiers. Moreover, Caputo et al. [22] used BC and another MRF-based method, statistical spin-glass MRV (SGMRV), to model the different regions within a mammogram. They followed it by a maximum a posteriori probability Bayesian classifier and were able to demonstrate that their proposed approach outperformed both the back propagation artificial neural network and the nearest neighbor classifier detection systems.

Several wavelet based MCs detection [14, 17–19, 21, 31] and enhancement [12, 13] schemes have been proposed in literature. Strickland and Hahn [21], for example, concluded that using an appropriate wavelet filter, one can easily detect and segment MCs within wavelet domain by thresholding the wavelet coefficients before the reconstruction process. Modeling MCs as highpass local anomalies, Wang and Karayiannis [14] also applied wavelet filtering, which is an elimination of an approximate wavelet subband and using detail subbands for reconstruction, they were able to detect MCs. Following [14], several studies used this wavelet filtering method for detecting suspected MCs [31] and to reduce false results [17]. Some studies demonstrated that least asymmetric Daubechies are more suitable for enhancement of the mammogram images such as in microcalcification detection [30] while other works demonstrated that the design of a spatial wavelet filter with high regularity is more successful in detecting microcalcifications than conventional wavelet filters such as the orthogonal Daubechies db4 [11]. Moreover, the none-stationary nature of mammogram image texture motivated many researchers to design wavelet transforms using adaptive filters which has been reported to be more efficient than fixed or none-adaptive FIR filter in the detection of low contrast MCs present in the denser breast tissue [31]. 

## 3. Segmentation Using Simplified Learning Bayesian Classifier

Learning machines for pattern recognition, such as artificial neural network, support vector machines and maximum a posteriori probability (MAP) classifiers consist of two phases: supervised learning and testing phases [19, 20, 22, 25, 26]. In the learning phase, a group of training samples that represents different objects or patterns to be extracted are selected manually to optimize the classifier's decision function while in the testing phase, the trained classifier is used to classify features contained in new data sets or the independent samples.

**Figure 2:**
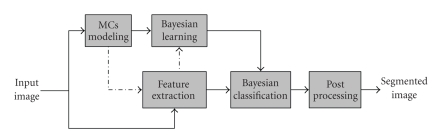
Segmentation using Bayesian learning.

Our proposed classification approach, Figure 2, follows the general structure of the classical learning machines but it uses a simplified learning stage denoted here as self-learning phase. Such a process can be relatively described as an unsupervised learning since it does not require the huge number of training samples of MCs to be extracted in advance form different data mammograms as the case of classical supervised learning [19, 22, 24] and instead it synthesizes these samples and use them as training data. 

In this work, detection is modeled as a two-class pattern recognition problem where the first class, *ω*_1_, is the clustered MCs group and the second class, *ω*_2_, is the healthy breast tissue. The proposed approach is described as follows.

**Figure 3:**
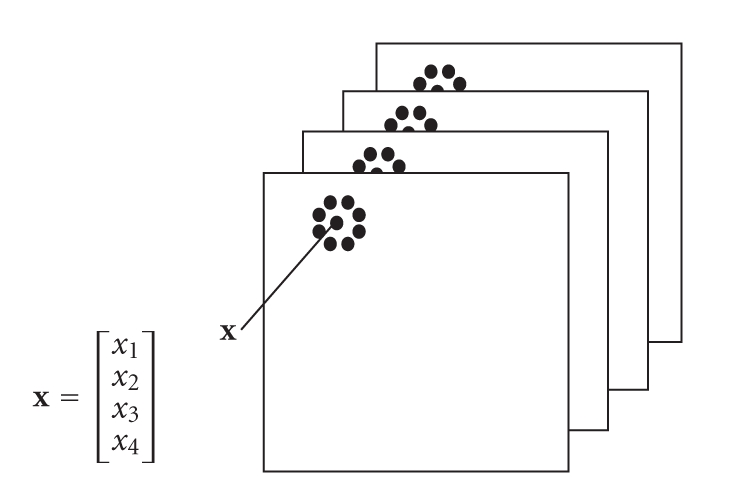
Composition of a pattern vector **x** using a four-image stack.

- *Modeling of microcalcifications*: the training samples of MCs class are synthesized by blending a synthetic model of MCs with a mammogram image. More details of this process will be explained in Section 3.1.
- *Feature extraction*: linear and none-linear transforms are used to extract three features of each pixel of a mammogram image. These three feature images along with the graylevel mammogram image are registered spatially to form a 4D pattern vector *x* = [*x*_1_ *x*_2_ *x*_3_ *x*_4_]^*T*^ of each class *ω*_*j*_ as shown in Figure 3.

In Figure 3, each pattern vector *x* is represented by a set of four components described as follows:

*x*_1_: Graylevel or image intensity
*x*_2_: Local maxima ranked using local histogram
*x*_3_: Spectral feature extracted using wavelet transform
*x*_4_: Singularity detection by detecting point discontinuity.

**Learning Phase**

The proposed learning process estimates the classifier's decision function parameters of each input mammogram, see Figure 2. Unlike the classical method which collects the training sets from different mammograms, the proposed approach extracts the training samples of different classes from the input mammogram itself as follows: for MCs class, it models the MCs and creates synthetic training samples of MCs class for that mammogram, the locations of these samples are identified using the binary model of the synthetic MCs. For the healthy breast tissue class, the training data are collected from two ROIs chosen randomly within the breast region.

(i) *Parameters estimation*: pattern recognition using stochastic BC is based on the estimation of the probability density function of each class. Assuming that the measured features of each class have a Gaussian probability distribution, the classifier's decision function can be computed as given in (4) requiring the estimation of the covariance matrix and the mean vector of each class. If the training set of each class is a sufficient statistically, one can efficiently estimate the distribution parameters (i.e., covariance matrix and mean vector) of each class.
 Further discussion of the parameter estimation for Bayesian classifier used in this work is presented in Section 3.4.
(ii) *Bayesian classification*: the optimized classifier is applied to perform a pixel based classification of the breast region into microcalcification and healthy tissue. In this work, the classification results are binary 0 or 1 and they are used to create a binary image by assigning a binary 1 to pixels classified as class, *ω*_1_ (or MCs), while a binary 0 is assigned to pixels classified as class *ω*_2_ (or healthy breast tissue).
(iii) *Post processing*: the purpose of this step is to reduce the false classifications and to improve the classification results through the integration of some of the physiological traits of breast tissue and clustered microcalcifications.

### 3.1. Construction of the Synthetic Microcalcifications

**Figure 4:**
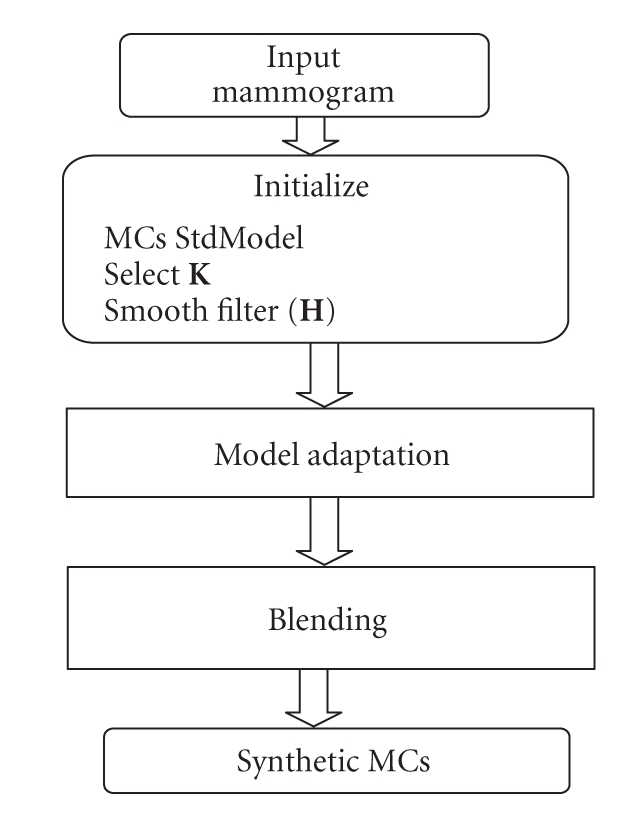
Construction of synthetic MCs.

In this work, we use a method proposed in [17] as an attempt to generate a model for real MCs as illustrated in Figure 4.

**Figure 5:**
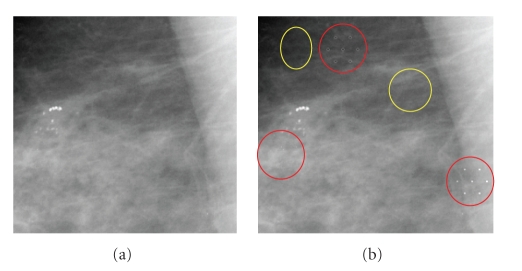
Synthesizing clustered microcalcifications: (a) original mammogram (b) mammogram with synthetic microcalcification, red circles correspond to the training samples of the microcalcifications while yellow circles represent the training samples for the normal (or healthy) tissue.

In this method, a new MC model is derived from the standard model (StdModel), a binary model of synthetic MCs, using input image and the modeling constant *K*. That is, each gray level value from synthetic pixels is assigned initially a fraction that is proportional to the constant *K* of its corresponding mammogram pixel and through a blending process, a hybrid image is created from the original mammogram and the modified MCs model. Such process is a pixel by pixel addition of the MCs model and mammogram followed by smoothing of the synthetic pixels using lowpass filter *H*, an example of the outcome of this scheme is shown in Figure 5. Our experimental results indicate that *K* should be chosen based on the statistics of the breast tissue of the mammogram such as the mean and variance of the breast tissue intensity values. 

MCs synthesis method presented in this work has a significant difference from the one introduced in [17]. This method has an explicit use of a modeling constant to control the synthesis of different types of MCs to ensure that a synthetic MC impersonates a real MC as much as possible. It is also worth noting that the purpose of using synthetic MCs in [17] was to provide a testing material for the detection scheme [17], while it is employed in this work as a detection tool and a control parameter of the scheme.

### 3.2. Feature Extraction and Formation of a Pattern Vector

This work uses the general structure of pattern recognition using Bayesian classifier which stacks and spatially registers a group of feature images. Each mammogram is represented by a stack of four images; (1) gray-level feature from original image, (2) feature image extracted using local maxima ranked using their local histogram, (3) highpass filtered image extracted using discrete wavelet transform, and (4) point singularity detected using Euclidian distance ED_8_.

#### 3.2.1. Highpass Filtering Using 2D Wavelet Transforms

Highpass filtering using discrete wavelet transform has proved to be a useful tool for detecting suspicious MCs [14, 17, 19]. In [14], the authors reported that orthogonal wavelet filters such db4 are more appropriate of detecting MCs since they have higher sensitivity to the presence of microcalcifications than other wavelet filters. Also, the spike-like behavior of db4 wavelet transform justifies the successful use of this wavelet filter for detecting specious MCs in [17, 19]. Therefore, we decided to employ db4 to extract the spectral features of MCs and to use this feature as one input feature of the Bayesian classifier.

**Figure 6:**
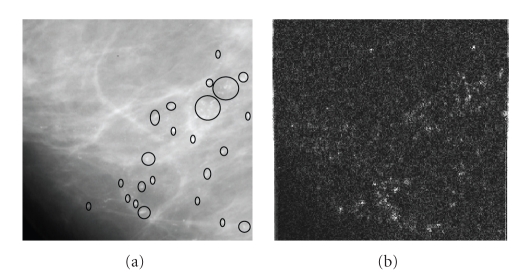
Highpass filtering using wavelet transform: (a) original mammogram region with MCs marked, (b) enhanced MCs (bright locations) obtained using 2-level DWT filtering process.

The basic idea behind this analysis is that, MCs represent highpass anomalies lay on a stationary lowpass background contributing to the detail subbands rather than to the coarse scale subbands of the wavelet multiresolution representation. In [14], the authors demonstrated that the features of MCs can be made more obvious after suppressing the background data, which is accomplished by eliminating the wavelet coefficients within coarse scale subband and reconstructing an image from detail subbands. An example of this process is illustrated in Figure 6.

#### 3.2.2. Feature Extraction Using Point Discontinuity

Spatially, microcalcifications appear as bright spots with various and mostly irregular shapes. Microcalcifications also appear in intensities that are higher than that of the surrounding healthy tissue.

**Figure 7:**
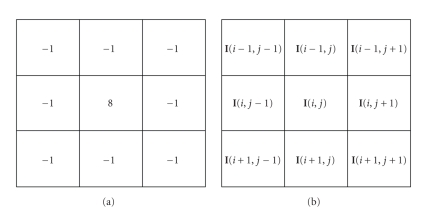
(a) Point detection kernel and (b) 3 × 3 block centered around pixel *I*(*i*, *j*).

**Figure 8:**
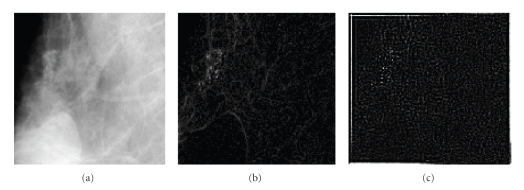
Feature extraction of microcalcifications: (a) original image, (b) texture features extracted using point discontinuity, and (c) spectral features extracted using wavelet based highpass filtering of image shown in (a).

Therefore, a pixel belong to a microcalcification region is expected to experience a larger gray-level difference from its local neighborhood than that of a healthy one. One approach to extract this type of singularity is by employing a point detection kernel as shown in Figure 7. In this work, the point singularity feature ED_8_ of each pixel is defined as the sum of the absolute difference of a pixel graylevel and those of its 8-neighboures. Example of this feature and other extracted features is demonstrated in Figure 8:

(9) $$\text{E}{{\text{D}}_{8}}_{ij}=\underset{k_{2}}{\sum }\;\underset{k_{1}}{\sum }\left|I_{i+k_{2},j+k_{1}}-I_{i,j}\right|,\;k_{1}=0,\pm 1,\;\;k_{2}^{}=0,\pm 1.\;$$

### 3.3. Learning Phase Using Synthetic Microcalcifications

Pattern recognition methods are in general supervised leaning machines [19, 20, 22–26], they partition the population data into training and validation sets. In such approaches, the training samples which are usually labeled manually, are employed to estimate the parameters of the classifier's decision function [19, 22].

Our proposed approach can be considered an unsupervised method and thus it does not require the training set of MCs class to be extracted from real mammograms as in the supervised manner. Instead, an adaptive and simple learning scheme is employed to estimate the classifier's parameters. The advantage of the proposed training scheme over the classical one is the use of synthetic MCs as training samples for the MCs class rather than using real MCs extracted from mammograms as practiced in the supervised methods [19]. 

Training stage starts by extracting four features from the breast tissue; these feature images are stacked to form a multidimensional feature vector of each pixel. The learning phase of the classifier is accomplished by blind, or unsupervised, selection of training data. Such selection is done by employing the binary MCs model to identify the training samples for MCs region while two distinct regions randomly selected within the mammogram are used to locate the healthy breast tissue candidates. A drawback of this random selection of the training samples of healthy breast tissue is the possibility that these regions may lay over breast areas that have low probability of developing malignant microcalcifications such as fatty or background regions. The negative impact of this practice can be eliminated by having a preprocessing step in which, the user mark the two regions within the glandular breast area or by employing a preprocessing step to identify the glandular breast region.

The learning process we propose has many advantages over the classical one; first, is the simplicity of the process with respect to the size of learning data, second, training samples of all classes, including the MCs, were selected manually in [19, 22, 24] while the training set of the significant class, which is the MCs, is synthetically constructed in this work. Another advantage of the proposed learning phase is that the training process of the classifier is adaptive to the breast tissue as the parameters of the classifier's decisions function are estimated using self-learning method based on the input data.

The proposed learning phase has two challenges. The first challenge occurs when the synthetic training samples are not statistically sufficient which may produce underestimation of the classifier's parameters. This limitation can be mostly attributed to the simplicity of the proposed modeling itself, that may have add some constraints on the ability to generate MCs training set of sufficient statistics from a single mammogram. The other challenge occurs when regions representing the training samples of healthy tissue include members of the other class (real MCs). While this work has not investigated the first problem, left it out as future work, the second challenge was addressed by using relatively large number of training samples of healthy (or background) class extracted from two different mammogram regions. The differencing in the sample size is significant due to the fact that mammogram texture is nonstationary and many samples of none-MCs class are available compared to the number of samples representing MCs that estimated to be no more than 1% of the whole mammogram. 

This work used about 4300 samples to represent the healthy (or background) class obtained from two distinct regions, which is about 50 times the size sample of MCs class. We investigated the effect of the sample size on the performance of the proposed detection scheme and the results indicated that a better detection can be obtained when two different regions used to extract the training samples of the healthy class than a single region. The results also indicated that the sample size of the healthy class must be larger, three times or more, than that of MCs class for better detection performance. 

### 3.4. Parameter Estimation of Bayesian Classifier

Assuming the two classes are equally likely and using the training pattern of both classes, our feature vectors, the decision function of the classifier is constructed by approximating the mean vector and covariance matrix [24] for each class as given by (4). The modeling constant, *K*, plays a significant role as it controls the appearance of synthetic MCs and their blending with the surrounding breast tissue. On approach that might be useful for selecting an appropriate value of the modeling constant prior to the training and classification stages is by measuring the difference between the corresponding components of the estimated mean vectors and the ratio of the corresponding diagonal entries (feature variances) of the estimated covariance matrices. Our investigation of both measures concluded that interpreting the mean difference is more obvious, that is easier to make a conclusion, and can be employed for better detection results.

Analyzing the interclass mean difference leads to identifying two cases; the first case occurs when a large value of the modeling constant is used, one that produces a large mean difference and leads to detect a single tone detail of the image, which might fail to detect the targeted MCs. This problem can be eliminated by adjusting the modeling constant to lower values before proceeding with training and segmentation stages. The second case occurs when a very small mean difference is used which decreases the discrimination power between classes and leads to an increase in false signals.

### 3.5. Segmentation via Bayesian Classifier

Testing the discrimination power of the classifier is usually accomplished by using the decision functions of the BC, (4), which is computed using the estimated covariance matrix and mean vector to classify an independent set of samples followed by computing the misclassification rate. The segmentation results are interpreted, form the classification results, as a target (microcalcification) and represented by a binary 1 and a nontarget (healthy tissue) represented by a binary 0. Both classes are assumed to be equally likely to occur.

## 4. Experimental Results

### 4.1. Mammogram Test Data

All mammograms used in this work are from a mini Mammographic database provided by Mammographic Image Analysis Society (MIAS) [32], which includes 23 cases with 28 MCs. Each mammogram from the database is a 1024 × 1024 pixels and with a spatial resolution of 200 *μ*m/pixel. These mammograms are already labeled with radiologist findings in terms of location and size of pathology results and tissue type which we found to be very useful when assessing experimental results.

### 4.2. Simulation Methods and Parameter Settings

The proposed scheme starts by modeling of the MCs in each mammogram as explained in Section 3.1. The most significant step of this process is the selection of the modeling constant *K*, a typical value of *K* can be chosen between 0.1 and 1. Then, spatial, textural, and spectral features of all pixels are extracted and used as inputs to the Bayesian classifier. The feature vector of each pixel is composed of the following: (1) brightness (or graylevel), (2) local maxima ranked based on the tail ratio of their local histogram estimated within 9 × 9 neighborhoods, (3) highpass filtered image obtained from suppressing coarse (or approximates) of the 2-level wavelet representation (db4 filters were used) and reconstructing an image from detail subbands, and (4) point singularity values computed using (9) as presented in Section 3.2.2. Stochastic Bayesian classifier optimized by a simplified self-learning phase is used to segment (or classify) all image pixels into MCs or healthy ones.

**Figure 9:**
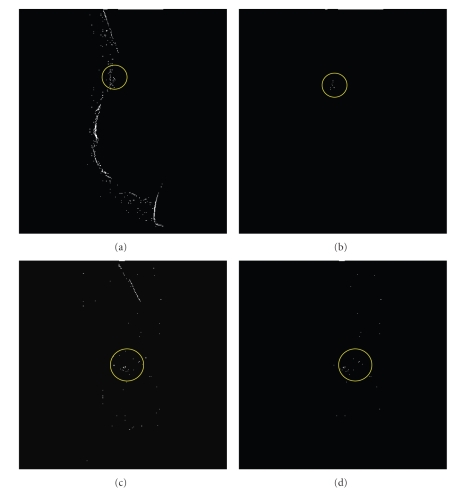
Improving the FP rate of the detection results using Otsu's thresholding: (a) and (c) results without post-processing, (b) and (d) post processing using Otsu's thresholding.

Experimental results demonstrate that applying this proposed approach to the whole mammogram without extraction or prior knowledge of breast region produces more false positive signals than those resulting from using breast region extracted form the whole mammogram. This result is illustrated in Figures 9(a) and 9(c) which also indicates that these false signals are mainly localized outside the breast region and can be significantly reduced using Otsu's thresholding [33]. Examples of this step results are demonstrated in Figures 9(b) and 9(d). In order to test the abilities of the proposed scheme for segmenting the whole mammogram and detecting the MCs, we used a simple thresholding scheme, Otsu's method, as a postprocessing instead of employing a prior breast region extraction or using some regional context within the detection process. Another advantage of using Otsu's thresholding as postprocessing was eliminating all misclassifications occurring along the breast border and outside the breast region. Such suppression process improved the detection performance by significantly reducing the overall number of false results, or misclassifications, while maintaining the detected MCs.

### 4.3. Experimental Results Analysis

Experimental results are assessed by computing the specificity and sensitivity parameters. This assessment would have been much more challenging without having the location and the size of true, real, MCs as documented by MIAS database [32]. Results indicate that synthesizing the training samples of MCs class and specifically the selection of the modeling constant *K* plays a significant role in the performance of the proposed classifier and the detection results. They also show that *K* values should be chosen based on statistics of the breast tissue characterized by the separation between the brightness of the region of MCs and that of the background tissue and the variance of the breast region. That is, the optimal *K* value is mostly correlated to the normalized mean difference (NMD) computed from the difference of the average graylevel (brightness) of the MCs and that of the of breast region.

Analyzing the breast regions of size 256 × 256 pixels extracted from each group indicates that a dense-glandular breast tissue has a larger intensity mean and variance than those of fatty breast tissue. Moreover, MCs that may be present in a fatty breast tissue have a larger NMD than those of MCs in dense-glandular breast tissue.

**Figure 10:**
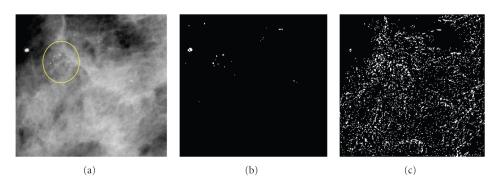
Detection results of MCs in a dense mammogram (mdb223) using different modeling constant *K* **:** (a) original mammogram (ROI of size 256 × 256 pixels, NMD = 0.05), (b) *K* = 0.25, (c) *K* = 0.1.

**Figure 11:**
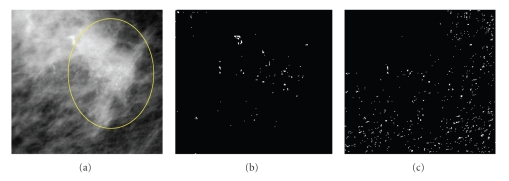
Detection results of MCs in fatty mammogram (mdb209) using different *K* **:** (a) original mammogram (ROI of size 256 × 256 pixels, NMD = 0.16), (b) *K* = 1, (c) *K* = 0.1.

Our results show that the modeling constant *K* can be adaptively chosen based on the type of breast tissue and the statistics of the breast region. Therefore, from all experimental simulations on mammogram ROIs of size 256 × 256 pixels, we found that small values of *K* such as 0.1–0.4 are suitable for detecting MCs occur within dense-glandular breast tissue while larger values of *K* such as 0.5–1 are more appropriate for MCs in fatty breast tissue. Such results are mostly due the fact that MCs present in denser mammogram tissue have lower local contrast than MCs occurring in fatty breast tissue. These results need to be further investigated on larger set of mammograms. Figure 10 illustrates the effect of the parameter *K* on the classification results, which shows that a large value of *K*  (*K* > 0.2) produces a detection of MCs with high specificity while low value of *K* as 0.1 or less leads to detection results with many false signals (or low specificity) as shown in Figure 10(c). On the other hand, large values of *K* are more appropriate (lower false signals) for detecting MCs appearing in a region that has high NMD and local variance as shown in Figure 11.

**Figure 12:**
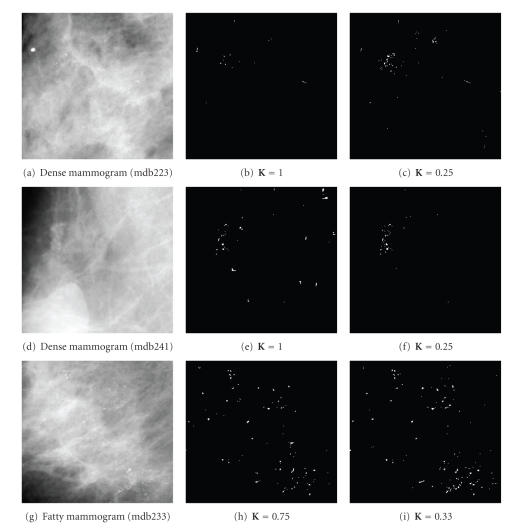
Examples of the detection results using different *K* values.

Results show that no optimal *K* value produces the best detection results (lowest FP and FN) for all test data but some values such as *K* = 0.2 produces the best TP and FP rates. Moreover, modeling MCs in dense breast tissue has shown more sensitivity to the value of *K* while the algorithm was more robust and allowed *K* to span a wider range for fatty breast tissue. Examples of these results are presented in Figure 12. Figures 12(c) and 12(f) show that low values of *K* between 0.2–0.25 are suitable for detecting MCs within both fatty and dense breast tissue. Furthermore, results indicated that MCs within Fatty breast tissue can be modeled and detected using wider range of modeling values with lower FP results at larger values of *K*, as demonstrated in Figures 11(b), 12(h), and 12(i). We believe that detection results can be further improved by fine tuning the selection of *K* if the breast region statistics were integrated into the algorithm. 

### 4.4. Performance Evaluation and Comparison

One approach to assess the performance of the detection scheme is by constructing a receiver operating characteristic (ROC) curve [3] which represents the false positive (FP) rates against the true positive (TP) rates obtained by varying a predefined control parameter such as *K*. Furthermore, other studies have used other metrics such as specificity and sensitivity parameters [3]. Specificity and sensitivity parameters are defined in terms of TP, TN, and FP as follows:

(10) $$\begin{array}{c} \text{Specificity}=\frac{\text{TN}}{\text{TN}+\text{FP}}\\ \text{Sensitivity}=\frac{\text{TP}}{\text{TP}+\text{FN}} \end{array}$$

where a true positive (TP) rate represents the probability of classifying a malignant tissue as target object while false positive (FP) refers to the probability of classifying healthy tissue as target one. Also, true negative (TN) rate is defined as the probability of classifying a healthy tissue as none-malignant and a false negative (FN) represents the case when a malignant tissue being classified as a healthy one.

In fact, MCs occur in mammogram in form of clusters rather than standalone. According to [2], a cluster of microcalcifications is defined as a group of three or more classification within a 1 cm^2^ area [2] which is equivalent to a block of 50 × 50 pixels in mini MIAS mammograms (digitized at 200 *μ*m edge resolution) Malignant calcifications also can only present in glandular breast tissue which is a fact that can also be used to eliminate any candidates (segmented as MCs) detected within dark regions. These physiological features are integrated in this work and mainly used in the computation of FP rates. Moreover, the difficulty in counting precisely the number of real calcifications within the region of true MCs forced us to count TP signal and report the sensitivity rates in a method similar to the one used in [10]. One reason for choosing TP per mammogram rather than per cluster is the nature of this proposed detection scheme, which applies a single model for segmenting all calcifications within the tested mammogram. Before proceeding any further with the performance evaluation, a summary of the definition of TP, FP, FN, and TN as used in this study ought to be stated.

- TP is identified by visual inspection of the detection results at image locations corresponds to the real annotated MCs region per the mini-MIAS database.
- FN is identified when a mammogram region of size 50 × 50 pixels that belongs to real annotated MCs per the database is detected as a background class.
- FP is identified when a healthy or a background region of size 50 × 50 pixels included three or more image locations detected as MCs class.
- TN is identified when a healthy or background region of size 50 × 50 pixels is detected as background class or it included a maximum oftwo locations of isolated MCs.

Using these definitions, the total number of TN, FP of a given output binary image is calculated by dividing the segmented image into nonoverlapping regions of size 50 × 50 pixels excluding the region of the real (actual) MCs as identified and labeled by the database.

Table 1 demonstrates the average specific-ity and sensitivity of the detection scheme obtained using BC optimized via self-learning methods.

Per Table 1, the best sensitivity (or TP rate) is about 91.3% at *K* = 0.2 and the corresponding specificity (FP rate) is at 98.6%. 

Although this proposed approach performs segmentation on a mammogram on a pixel level, it does estimate FP rates using region basis by utilizing physiological characteristics of clustered MCs, which is different form the previous work reported in [19]. This fact makes it unreasonable to attempt to have a direct numerical comparison between our detection results and those obtained in [19, 20]. However, in [19], the authors reported their results using 87 × 87 block sizes and used the total number of the true MC samples to be 25 MC regions. 

To ensure unbiased comparison with results reported in the literature, we decided to evaluate the performance of the detection results form this study in a similar manner. We used the same block size and counted FP to be the case when an MC is detected.

However, TP is still evaluated by visually inspecting the detection results and comparing it with the real MCs as reported by the database. Table 2 compared the specificity and the sensitivity or (TP and FP rates) of the proposed scheme with relevant works, which indicates that this proposed scheme produces lower TP and higher FP rates (or lower specificity) compared to those from [19] but better than [20].

## 5. Discussion and Conclusions

In this paper, we proposed and implemented a new approach using stochastic Bayesian classifier for segmenting a digital mammogram for the detection of microcalcification clusters. The proposed scheme models the image segmentation task as a two-class pattern recognition problem. This new framework accomplishes the learning phase of the classifier using a simple self-learning approach which synthesizes the training samples of MCs class in each mammogram. Each image pixel, during both the learning and testing phases, is modeled using a four-feature vector extracted using spatial, statistical, and spectral via wavelet filtering methods. The proposed scheme was tested using 23 mammograms from mini-MIAS database. Results demonstrated that synthetic patterns can be employed to simplify the supervised Bayesian learning for MCs detection, which produces moderate detection performance. The relatively high FP and low TP rates can be related to the simplicity of MCs modeling used in this study as well as applying the detection scheme to the whole mammogram as opposed to regions continuing only breast tissue. Other image processing techniques can be used, such as fuzzy K-mean clustering, to extract glandular breast regions and thus eliminating many of the misclassifications (FP) that are resulting at the breast borders and radiographic markers. 

Another improvement may be suggested is to use a more sophisticated modeling of healthy breast tissue and MC regions which should be useful to overcome some of the classifier's parameters' estimation effects on results.

## Figures and Tables

**Table 1:** Detection results using self-learning BC.

**K**	0.17	**0.2**	0.25	0.33	0.5	0.75	1.0
Specificity %	96.9	**98.6**	99.2	99.2	98.9	98.4	98.1
Sensitivity %	95.7	**91.3**	78.3	69.5	56.5	52.2	56.5

**Table 2:** Comparison with related work.

Study	Database	Sensitivity %	Specificity %	FP/image
Proposed I*	**MIAS**	**91.3**	**98.6**	6.15
Proposed II*	MIAS	91.3	96.4	5.1
Yu et al. [19] (BC)	MIAS	92	97.8	0.75
Yu et al. [19] (BPNN)	MIAS	92	98.9	1.5
Huang and Yu. [20] (SVM)	MIAS	76	88	NA
Huang and Yu. [20] (BPNN)	MIAS	72.15	78.4	NA

*Proposed I and II obtained using region size of 50 × 50 and 87 × 87, respectively.
